# Neuroprotective properties of extra virgin olive oil polyphenols in Alzheimer’s disease: a multi-target mechanistic review

**DOI:** 10.3389/fnut.2025.1736633

**Published:** 2025-11-27

**Authors:** Lin Wei, Zhenmin Li, Mingqin Shi, Wu Song, Zhanguo Teng, Chi Zhang

**Affiliations:** 1 School of Basic Medical Sciences, Changchun University of Chinese Medicine, Changchun, Jilin, China; 2 College of Chinese Medicine, Changchun University of Chinese Medicine, Changchun, Jilin, China; 3 First Clinical Medical College, Yunnan University of Chinese Medicine, Kunming, Yunnan, China; 4 Affiliated Hospital Nongan Branch, Changchun University of Chinese Medicine, Changchun, Jilin, China

**Keywords:** Alzheimer’s disease, extra virgin olive oil, polyphenols, neuroprotective effects, multi-target

## Abstract

Alzheimer’s disease (AD) is a complex neurodegenerative disorder characterized by β-amyloid (Aβ) deposition, hyperphosphorylated tau protein, neuroinflammation, and mitochondrial dysfunction. The limited efficacy of single-target pharmacological strategies has spurred interest in multi-target therapeutic approaches. Extra virgin olive oil (EVOO), rich in diverse polyphenolic compounds, has emerged as a promising source of such multi-target neuroprotective agents. This review systematically elucidates the mechanisms of key EVOO polyphenols-hydroxytyrosol, oleuropein, tyrosol, verbascoside, oleocanthal, and ligustroside-in combating AD pathology. We highlight the growing body of evidence demonstrating that these polyphenols can synergistically inhibit the aggregation of Aβ and tau, mitigate neuroinflammation, restore mitochondrial function, reduce oxidative stress, and promote neurogenesis. Preclinical studies in cellular and animal models of AD consistently show that EVOO polyphenols can ameliorate cognitive deficits and pathological hallmarks. Future research should focus on validating these benefits in animals and clinical trials and developing optimized formulations for clinical application. In conclusion, the bioactive polyphenols in EVOO present a compelling multi-targeted therapeutic strategy with significant potential to delay the progression of AD by concurrently modulating multiple key pathological pathways.

## 1 Introduction

Cognitive and functional decline in Alzheimer’s disease (AD) follows a characteristic trajectory with specific onset, progression rate, and neuropathology (1). Alois Alzheimer first reported that extracellular plaques and intracellular neurofibrillary tangles (NFTs) comprise the main pathological hallmarks of AD in his 1907 paper (2). In recent years, human enzymes have been a focus of research across many scientific fields. Traditionally, AD is tackled with drugs like acetylcholinesterase inhibitors (e.g., donepezil, galantamine, rivastigmine) (3–5) and the N-Methyl-D-Aspartate (NMDA) receptor allosteric modulator, memantine (6–8). Though these drugs have therapeutic effects, they also have significant drawbacks. According to a recent study that contrasts the former dogma, which believes that targeting Aβ would benefit the majority of AD patients, it is now believed that anti-Aβ monoclonal antibodies will not offer a magic bullet (9).

Consequently, there is a need to seek alternative treatments for AD, which is becoming more and more of a current focus. Medicinal herbs are both edible and medicinal (10, 11). Hence, they cause a substantial reduction in the side effects of medicines and drugs. On the other hand, most chemically synthesized drugs are designed to be single-target agents with a working hypothesis (12). As a result, if the hypothesis turns out to be false, the effectiveness of these drugs often falls apart. Unlike synthetic drugs, naturally made drugs act on multiple targets to give multiple effects. This ability to influence multiple targets makes them particularly useful for the treatment of diseases whose origins are not fully understood, like AD (13–17).

**Figure 1:**
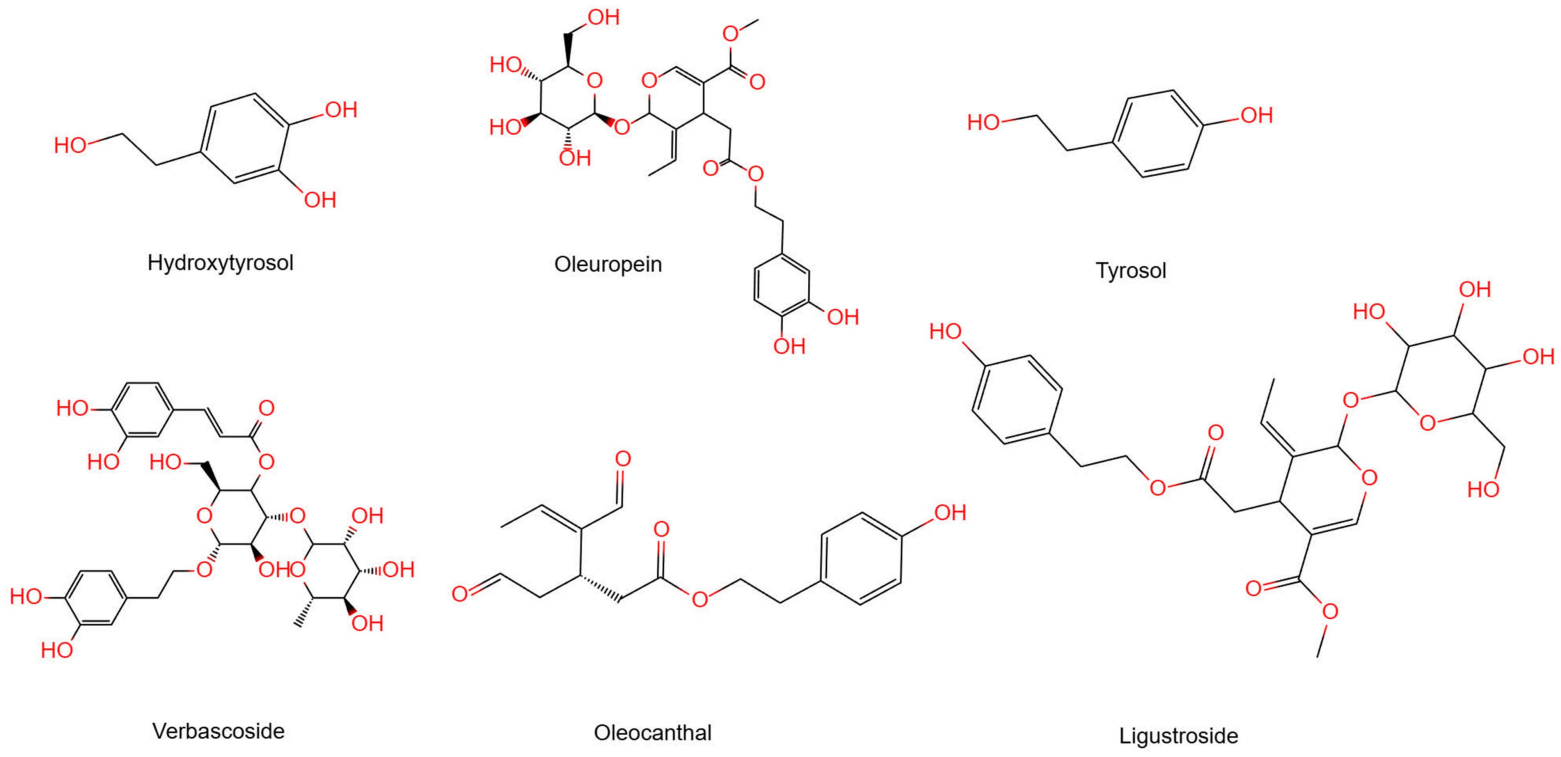
The structure of chemical compounds in olive oil.

Polyphenols are organic compounds that can be natural, synthetic, or semi-synthetic, having more than one phenolic group (18). This means polyphenols usually consist of one or more aromatic rings with hydroxyl groups. Research shows that natural plant polyphenols have positive effects on human health over the years. The chemical composition of olive oil will depend on the extraction method used to obtain it from the fruit. Refined olive oils are devoid of vitamins, polyphenols, phytosterols, and other low-weight natural constituents. Unlike other varieties of olive oil, EVOO is more expensive due to its lower yield, but it contains the most polyphenols (19–22). The unique phenolic compounds present in olive oil, especially the phenolic alcohols hydroxytyrosol (3,4-DHPEA) and tyrosol (p-HPEA), as well as their respective secoiridoid derivatives, mainly 3,4-DHPEA-EA (oleuropein aglycon), p-HPEA-EA (ligstroside aglycon), 3,4-DHPEA-EDA, p-HPEA-EDA (oleocanthal), and oleuropein (23), have recently been attributed the chemopreventive potential of olive oil (Figure 1). The cancer-fighting ability of olive oil is connected to the antioxidant nature of its phenolic and polyphenolic constituents, which help in neutralizing free radicals and reactive oxygen species. The compounds oleuropein, tyrosol, hydroxytyrosol, verbascoside, ligustroside, and dimethyl europein protect against coronary artery disease (24–26) and cancer (27, 28). They also possess antimicrobial and antiviral properties (29, 30). The antioxidant and antiatherogenic effects of oleuropein and hydroxytyrosol (polyphenols from olive oil) are well established (31, 32).

**Figure 2:**
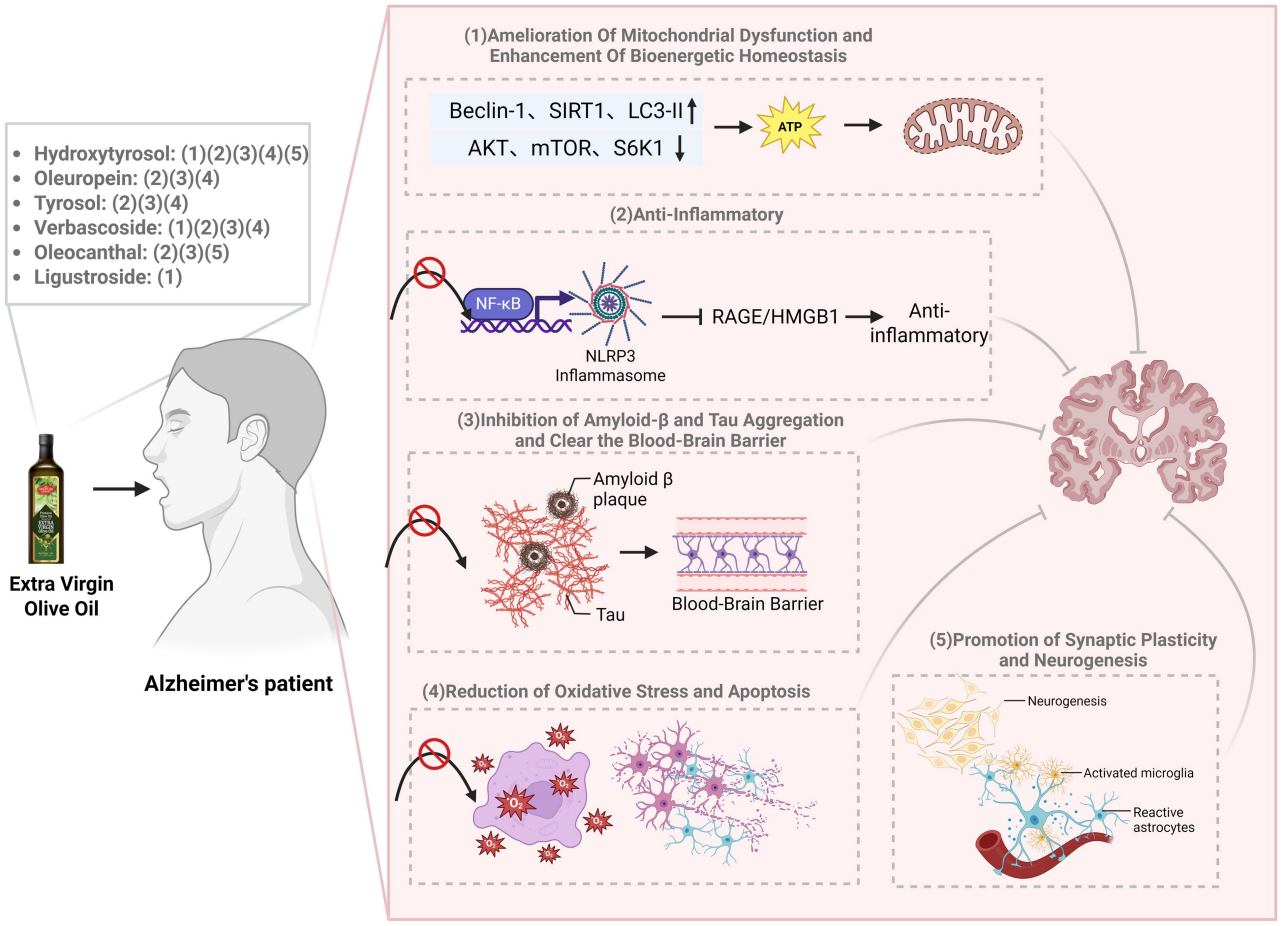
The mechanism by which EVOO exerts neuroprotective effects on Alzheimer’s disease. EVOO polyphenols exert synergistic neuroprotection through pleiotropic mechanisms. Key findings include: (1) amelioration of mitochondrial dysfunction and enhancement of bioenergetic homeostasis; (2) potent anti-inflammatory effects via suppression of NF-κB and NLRP3 inflammasome signaling; (3) inhibition of amyloid-β and tau aggregation, alongside promotion of amyloid clearance across the blood–brain barrier; (4) reduction of oxidative stress and apoptosis; and (5) promotion of synaptic plasticity and neurogenesis.

Despite the established neuroprotective potential of individual EVOO polyphenols in preclinical models, a systematic review synthesizing their multi-target, synergistic mechanisms against the complex pathology of AD are lacking. This review aims to fill this gap by critically evaluating the mechanistic evidence for the most prominent EVOO polyphenols-hydroxytyrosol, oleuropein, tyrosol, verbascoside, oleocanthal, and ligustroside-in targeting key AD pathways, including Aβ and tau aggregation, neuroinflammation, oxidative stress, and mitochondrial dysfunction (Figure 2). Furthermore, we discuss the challenges in clinical translation and the relevance of existing human studies (see Table 1).

**Table 1:** Extra virgin olive oil polyphenols for treating AD.

Number	Name	Research object	Modeling method	Mechanism	Reference
1	Hydroxytyrosol	VAFs	/	Increase LC3 and Beclin1, TNF-α, SIRT1, inhibit Akt/mTOR, regulate autophagy in VAFs, and suppress inflammatory response in VAFs.	(85)
2	Hydroxytyrosol	Mice	/	Improve spatial working memory and restore brain ATP levels.	(37)
3	Hydroxytyrosol	Mice	*Btg1-null*	Increase the survival rate of newly generated neurons, reduce apoptotic cell death in the dentate gyrus, and weaken markers of cellular aging (including lipofuscin accumulation and Iba1 immunoreactivity).	(38)
4	Hydroxytyrosol	PC12	α-Syn	Increase the expression of SIRT-2, HO-1, Hsp70, and SIRT-2.	(41)
5	Hydroxytyrosol	Mice	Soluble oligomeric amyloid β1-42 plus ibotenic acid (oA42i)	Enhance spatial cognitive ability, inhibition of JNK - and p38 MAPK, activation of ERK–MAPK/RSK2, PI3K/Akt1, and JAK2/STAT3, while preserving mitochondrial superstructure, upregulation of genes involved in regulating survival and memory function.	(42)
6	Hydroxytyrosol	Mice	APP/PS1	Improve neuronal apoptosis and reduced levels of inflammatory cytokines, and improve the escape latency, escape distance, and platform crossing times of AD mice in the water maze test.	(86)
7	Oleuropein	Mice	5xFAD	Inhibit NF-κB, NLRP3 inflammasome, and RAGE/HMGB1 to alleviate neuroinflammation. Reduce the production of A β, enhance BBB integrity and function, and lower the total A β level in the brain, thereby improving memory function.	(48)
8	Oleuropein	Mice	TgCRND8	Reduce the area and quantity of A ß 42 and pE-3A ß plaques in the cortex, decrease the area and quantity of plaques in the cortex and hippocampus, and improve cognitive function.	(49)
9	Oleuropein	Rat	OLE and Aβ42	Reduce soluble A β oligomer levels, alleviate neuronal loss, and decrease neuroinflammatory responses.	(54)
10	Oleuropein	RIN-5F	hIAPP	Interference with amyloid protein aggregation leads to skipping the formation of toxic precursor fiber aggregates.	(55)
11	Oleuropein	*C. elegans*	CL2006 and CL4176	Reduce Aβ plaque deposition and decrease the abundance of toxic Aβ oligomers.	(56)
12	Oleuropein	*Escherichia coli* BL21 (DE3) cells	pET28b/α-syn plasmid	Obstruct the binding of alpha synuclein aggregates to cell membrane components and prevent the resulting oxidative damage to cells, reduce cytotoxicity.	(57)
13	Oleuropein	Mice	TgCRND8	Interference with the aggregation of A ß 42 and pE3-A ß. Activate neuronal autophagy, increase histone 3 and 4 acetylation, reduce histone deacetylase 2 expression, and improve synaptic function.	(58)
14	Oleuropein	Rat	Morphine	Improve spatial learning and memory impairments. Reduce cell atrophy and oxidative stress levels in the CA1 region of the rat hippocampus.	(59)
15	Tyrosol	Mice	5XFAD	Increase spike protein, reduce 4-hydroxy-2-nonenal immunoreactivity in hippocampal CA3 region, alleviate spatial memory impairment in AD mice.	(64)
16	Tyrosol	N2A cells	Aβ (25-35)	Reduce Aβ - induced cytotoxicity, decrease NF-κB activation, and inhibit nuclear translocation of NF-κB subunits.	(65)
17	Tyrosol	Mice	Scopolamine	Reduce the levels of acetylcholinesterase, A β 1–42, APP, and MDA, and increase SOD, CAT, Nrf2/HO-1.	(66)
18	Verbascoside	Mice	APP/PS1	Block the activation of microglia and astrocytes, inhibit IL-1β and IL-6, enhancing IL-4, IL-10, and TGF-β, and inhibit the phosphorylation of IKK α + β, IκBα, and NF-κB-p65.	(67)
19	Verbascoside	Mice	APP/PS1	Enhance the learning and memory abilities of mice. Reduced expression of pro-inflammatory M1 microglial markers (CD11b, iNOS, and IL-1β); The increased expression of M2 microglial cell markers (Arg-1 and TGF-β1) promotes the transition of microglia from M1 pro-inflammatory phenotype to M2 anti-inflammatory phenotype. Downregulate the expression of TLR4/NF-κB and upregulate the expression of synaptic proteins.	(69)
20	Verbascoside	Mice	D-galactose and AlCl	The number of neurons and Nissl bodies in the hippocampus increases. Decreased levels of nitric oxide content, nitric oxide synthase activity, and caspase-3 protein expression.	(70)
21	Verbascoside	Mice	D-galactose and AlCl	Reduce the escape latency of mice searching for platforms and increase the number of platform crossings. Increase the expression of nerve growth factor and promycin receptor kinase A in the hippocampus.	(71)
22	Verbascoside	Mice	APP/PS1	Improve memory and cognition in mice. Inhibit cell atrophy, reduce A β deposition, decrease the formation of neurofibrillary tangles caused by excessive phosphorylation of tau protein, and downregulate the expression of neurotrophic factors.	(72)
23	Verbascoside	U251 cells	Aβ1-42-damaged	Enhance cell viability, inhibit cell apoptosis, reduce calcium accumulation and intracellular reactive oxygen species concentration, and improve mitochondrial and ER morphology.	(72)
24	Verbascoside	PC12	Aβ1-42-damaged	Promote the influx of extracellular Ca^2+^. Upregulate the expression of p-CaMKII. and downstream vesicle associated proteins, reduce the activity of AChE, and prolong the action time of ACh in synaptic cleft.	(73)
25	Oleocanthal	Mice	5xFAD	Inhibit NF-κB, reduce the activation of NLRP3 inflammasome, lower brain A β levels and neuroinflammation. Inhibit RAGE/HMGB1 pathway receptors.	(77)
26	Oleocanthal	Neurons and Astrocytes	A β oligomer	Reduce the expression of synaptic proteins SNAP-25 and PSD-95, and increase the expression of GLT1 and GLUT1. Reduce IL-6 and downregulate GFAP.	(79)
27	Oleocanthal	Mice	TgSwDI	Reduce the protein load of Haidian powder samples. Enhance the clearance rate of BBB. Increase the expression of P-glycoprotein and LRP1, and activate the ApoE dependent amyloid clearance pathway. Reduce astrocyte activation and IL-1 β levels.	(80)
28	Oleocanthal	Mice	/	Enhance the clearance rate of (125) I-A β 40 from the brain, increase the expression and activity of P-gp and LRP1.	(81)
29	Oleocanthal	Mice	TgSwDI	Inhibit NACHT, LRR, and NLRP3 inflammasomes to reduce neuroinflammation and activate AMPK/ULK1 pathway to induce autophagy, thereby restore BBB function and reducing AD related pathological damage.	(83)
30	Oleocanthal	Mice	5xFAD	Reduce beta load, upregulate myosin, enhance brain barrier integrity, and reduce neuroinflammation.	(84)

## 2 Hydroxytyrosol

Hydroxytyrosol (HTyr) is the main phenolic molecule found in EVOO. It is a low molecular-weight phenotype with a catechol group. This chemical structure is responsible for its high bioactivity, which has antioxidant, cardioprotective, anticancer, anti-inflammatory, and notably neuroprotective effects (33–35).

HTyr is characterized by a catechol group that confers potent antioxidant, anti-inflammatory, and neuroprotective properties. The ability of HTyr to boost mitochondrial quality control is primarily responsible for its neuroprotective efficacy. It increases the nutritional absorption by improving its functionality in the intestine. It increases nitric oxide release, thereby improving blood flow to the digestive organs. It induces Epidermal Growth Factor (EGF) release and inhibits apoptosis. It also promotes cell proliferation through the epidermal growth factor receptor. The intracellular ATP levels are increased by HTyr and olive polyphenols in SH-SY5Y-APP695 cells (AD model) (36). In a similar vein, HTyr normalizes cerebral ATP production as well as enhances the activities of different electron transport chain components (NADH reductase, cytochrome c oxidase) and citrate synthase in 12-month-old mice with mitochondrial dysfunction. The metabolic recovery resulted in increased SIRT1 indications and concentration of CREB, Gap43 and GPX-1, which significantly improves spatial working memory (37).

HTyr worked positively on the hippocampal neurogenesis, thus preventing age decline in neurogenesis decline in case of neurodegenerative diseases. In adult, aged, and *Btg1* knockout mice, HTyr treatment reduces apoptotic death of newborn neurons in the dentate gyrus, thus enhancing cell survival. According to (38), it also reduces markers of cellular senescence like lipofuscin aggregation and Iba1 immunoreactivity, proposing a role in the modulation of glial activation and oxidative stress. Another important mechanism is the inhibition of pathological protein aggregation. HTyr and oleuropein aglycone (OLE) inhibit Tau fibrillization, thereby reducing neurofibrillary tangle (NFT) formation and associated neuronal and glial pathology (39). HTyr metabolites also inhibit amyloid-β deposition in cellular models (40). In neuronal PC12 cells, these strong experiments reveal that HTyr suppresses the aberrant aggregation of α-synuclein, a protein that plays a key role in synucleinopathies. Also, it upregulates the deacetylase SIRT2, which possibly facilitates the clearance of misfolded proteins (41). These helpful benefits do result in cognitive improvement. Mice’s brain injections of Aβ1-42 caused loss of memory, but HTyr, in essence, helps mice to rescue their memory and control the apoptosis process (42). In APP/PS1 (Amyloid Precursor Protein/Presenilin 1) transgenic mice, a well-characterized transgenic model of AD, HTyr treatment improves performance in the Morris water maze test, reduces cortical and hippocampal apoptosis, and restores synaptic integrity (42).

HTyr confers neuroprotection through a multitude of actions, including improving mitochondrial function, enhancing neurogenesis, and inhibiting inflammation, apoptosis, and protein aggregation. It is ability to target multiple nodes of the neurodegenerative cascade underscores its potential as a polypharmacological agent for AD and other dementias.

## 3 Oleuropein

Oleuropein (OLE) is the major phenolic found in olives. It is a kind of secoiridoid phenolic compound, which is made of three structural building blocks: HTyr, elenolic acid and glucose. It shows a wide variety of biological activities such as antioxidant, anti-hypertensive, and anti-inflammatory activities (43–46).

The neuroprotective effects of OLE are mediated through various mechanisms. The processes involve the induction of autophagy, activation of antioxidant capacity in various brain areas, and inhibition of neuroinflammation by inactivating microglia and astrocytes. Inactivation diminishes a greatly excessive release of pro-inflammatory mediators (47). It seems that people who eat OLE regularly have a lower risk of developing strokes, AD, Parkinson’s, and other neurological disorders. These advantages are supported by animal studies. Starting at 3 months of age, mice were fed a diet enriched in oleuropein (695 μg/kg b.w. per day) for 3 months. According to overall findings, OLE inhibits activation of the RAGE/HMGB1 pathway and NOD-like receptor thermal protein domain associated protein 3 (NLRP3) inflammasome and NF-κB pathway, thus reducing neuroinflammation. Furthermore, OLE decreased total Aβ levels in the brain. This is due to enhanced clearance as well as decreased Aβ production along with superior blood-brain barrier (BBB) integrity and functioning. Due to these alterations, these improvements all lead to better memory performance (48). Supplementation of OLE (12.5 mg/kg/day) plus a polyphenol mixture improved cognitive capacity in transgenic (Tg) mice according to a different study (*p* < 0.0001). OLE significantly decreased cortical Aβ42 levels as well as areas and numbers of pyroglutamate-modified Aβ3–42 (pE-3Aβ), a highly pathogenic and aggregation-prone Aβ species (49).

A crucial element of OLE’s activity is its direct anti-aggregation action. The OLE aglycone exhibits multiple actions that counteracts the aggregation of the amyloid protein and mitigate its toxicity through various mechanisms; these include the processing of the amyloid precursor protein, the aggregation of the amyloid beta peptide and the tau, impaired autophagy, and neuroinflammation that have therapeutic effects in AD (21, 50–53). OLE, a phenolic compound found in extra virgin olive oil (EVOO), inhibits the toxicity and aggregation of Aβ42 in a rat model. The levels of soluble Aβ oligomers were significantly reduced as a result of OLE co-injection. Oleuropein prevents the cytotoxic effects of amyloid in cells when present (54). Analyses have shown that when amyloid aggregates form in the presence of oleuropein, they interact less with cell membranes as oleuropein alters the aggregation pathway away from the formation of toxic pre-fibrillar aggregates (55).

In transgenic *Caenorhabditis elegans* models of AD (CL2006 and CL4176), which express human Aβ and exhibit paralysis as a phenotypic readout, OLE treatment reduced Aβ deposition and delayed paralysis. In CL2006 worms, OLE treatment reduced Aβ deposition and oligomer form, delayed paralysis, and increased lifespan. The CL4176 worms only showed the protective effect of OLE when it was given before the induction of Aβ expression. These effects, which depend upon dose, do not seem (56). Moreover, OleA was also found to reduce cell toxicity by blocking the attachment of α-synuclein aggregates to cell membrane components, which cuts down on secondary oxidative damage to the cells (57). The OLE aglycone has anti-aggregation activity against the aggregation of both aggregation of Aβ42 and pE3-Aβ, together with reducing the expression of the enzyme, and interferes with pE3-Aβ formation due to glutaminyl cyclase. Furthermore, neuron autophagy was activated in the presence of this compound, which increased histone acetylation. This effect was related to downregulation of HDAC2, and a remarkable improvement in synaptic function was observed in mice showing advanced pathology (58). In another study, treatment of oleuropein (15 and 30 mg/kg) significantly improves spatial learning and memory in morphine given animals and reduces cell atrophy and oxidative stress in the hippocampus (59).

So, OLE and supplementing the diet can be a good promise to stop and/or slow the progression of AD. Oleuropein is a natural antioxidant compound that shows considerable neuroprotective potential. Preclinical evidence strongly suggests the ability of OLE to combat certain chalk marks of AD, PD, and other similar-related ailments, improving cognitive activity and synaptic function. The findings demonstrate oleuropein’s potential as a valuable candidate for further translational research and dietary intervention strategies to slow down the progression of neurodegenerative diseases.

## 4 Tyrosol

Tyrosol is a bioactive phenolic compound found in high concentrations in natural products such as olive oil and wine. It has a large variety of biological functions that include antioxidant, anti-inflammatory, cardioprotective, and neuroprotective effects (60–62). Increasing evidence from preclinical studies suggests that it may help in preventing neurodegenerative pathology.

Animal studies have shown that treatment with tyrosol significantly improves cognitive deficits *in vivo*. In the hippocampus, tyrosol reduced oxidative stress markers (ROS and MDA) and enhanced activities of antioxidant enzymes (GPX and SOD) in PMD rats. Further, concentrations of brain-derived neurotrophic factor (BDNF) and monoamine neurotransmitters (5-HT, DA, and NE) were significantly increased here (63). In AD models, a study reported that oral tyrosol decreased spatial memory impairment as measured by the Barnes maze test and decreased oxidative damage in the CA3 region of the hippocampus, a subregion critical for spatial memory and learning (64). Tyrosol has significant anti-amyloidogenic properties at the molecular level. Key contributors to AD pathogenesis, soluble Aβ oligomers, trigger toxicity to cells and synapses. Primary neurons showed a significant inhibition of caspase-3 by tyrosol induced by Aβ Oligomers (AβO). Co-treatment with tyrosol or its metabolite HTyr resulted in the decrease of Aβ-induced cytotoxicity in neuroblastoma N2a cells (65). These results demonstrate that tyrosol protects against Aβ toxicity by counteracting oxidative stress and modulating inflammation.

Notably, extracts rich in tyrosol show strong neuroprotective efficacy. *Rhodiola sachalinensis*, which is rich in tyrosol, improved cognitive performance in scopolamine-induced amnesia models. Taking *R. sachalinensis* improved performance in Y-maze, passive avoidance, and water maze tests. This was also accompanied by reduced activity of acetylcholinesterase, increased activity of antioxidant enzymes (SOD and CAT), and downregulation of the expression of Aβ_₁–₄₂_ and APP (66).

Together, these studies suggest that tyrosol protects the brain through many different effects, including reducing oxidative stress, boosting brain cell growth signals, protecting against Aβ damage, and controlling cell death and inflammation. Increasing evidence from preclinical studies suggests that it may help in preventing neurodegenerative pathology.

## 5 Verbascoside

Verbascoside (VB) has emerged as a promising neuroprotective agent in experimental models of AD, demonstrating multiple mechanisms countering key pathological processes, including neuroinflammation, oxidative stress, synaptic dysfunction, and protein misfolding (67, 68).

One of the mechanisms through which VB exerts its beneficial effects is the modulation of neuroimmune response. Under APP/PS1 transgenic mice (the most popular Alzheimer’s disease model), the treatment with VB drastically inhibited the activation of microglia and astrocytes. This induced a shift from the pro-inflammatory M1 phenotype, as indicated by decreased expression of CD11b, iNOS, and IL-1β, to the anti-inflammatory M2 phenotype, as indicated by increased levels of Arg-1 and TGF-β1. This shift is largely mediated by suppression of the NF-κB signaling pathway. The administration of VB significantly decreased the phosphorylation levels of IKKα and IKKβ, IκBα, and NF-κB-p65. Furthermore, VB reduced the nuclear translocation of NF-κB-p65 *in vivo* and *in vitro*. This suggests that VB has a potent anti-inflammatory activity (67, 69).

At the same time, VB treatment significantly improves cognitive performance and pathology. In rodent models like APP/PS1 and D-galactose/AlCl3-induced aging mice, VB improved learning and memory functions. According to Morris water maze and step-down tests, VB reduced escape latency, increased the number of platform crossings, and decreased errors (70, 71). VB reduced the deposition of Aβ plaques and attenuated the formation of neurofibrillary tangles (NFTs) composed of hyperphosphorylated tau, which happens due to neuronal atrophy. Also, correlation of structural, biochemical functional benefits was noted. Also, correlation of structural biochemical functional benefits was noted. These happened due to neuronal atrophy and enhanced density of Nissl bodies and neurons of the hippocampus (70, 72). Moreover, VB was found to suppress 4-hydroxynonenal biomarkers and caspase-3 activity (70, 72). Subsequently, these findings indicate that VB downregulated apoptosis. At the cell level, VB protected against toxicity from Aβ. In U251 and PC12 cells damaged by Aβ_₁–₄₂_, VB improved cell viability, suppressed apoptosis, inhibited cytosolic calcium accumulation and reactive oxygen species, and modified mitochondrial and endoplasmic reticulum (72, 73). Additionally, VB also changed synaptic function by inducing extracellular Ca^2+^ influx through L-type voltage-gated channels that activated the CaMKII pathway, upregulated synaptic vesicle-associated proteins and inhibited the activity of acetylcholinesterase (73). Aside from these mechanisms, VB and its esterified derivative verbascoside penta propionate (VPP) inhibit Aβ aggregation dynamics and its cytotoxicity with no metal ion, indicating the involvement of another mechanism in its neuroprotection profile (74).

Taken together, the evidence from experiments points to VB being a multi-target neuroprotective agent that acts effectively in diverse AD models. VB is a huge neuroinflammatory agent. It significantly reduces the activity of microglia and astrocytes at a cellular level. There is a change from M1 to M2, which means from an inflammatory to an anti-inflammatory environment. It accomplishes this through reduction of the NF-κB signaling pathway. At the same time, VB improves cognitive impairments and decreases characteristic illnesses of the nervous system, including Aβ deposition, tau hyperphosphorylation, and damage caused by oxidative stress. Also, it enhances neuronal resilience by reducing apoptosis, stabilizing calcium homeostasis, and preserving mitochondrial integrity while benefiting synaptic function through Ca^2+^-mediated signaling and cholinergic boosting. Finally, VB and VPP inhibit Aβ aggregation, complementing the metal ion-binding mechanism, through their direct action against Aβ. Together, these attributes position VB as a promising multifaceted candidate for slowing AD progression.

## 6 Oleocanthal and Ligustroside

The presence of oleocanthal and ligustroside in extra virgin olive oil (EVOO) is increasingly being studied for their neuroprotective effects in the model of Alzheimer’s disease (AD). The OC has been developed quite well in many experimental systems. But ligustroside is a candidate that has promise, but has not yet been evaluated.

### 6.1 Multifaceted mechanisms of Oleocanthal

Oleocanthal (OC) exhibits an extraordinary ability to target multiple key pathological pathways involved in AD (75–78). The anti-inflammatory properties constitute a main mechanism of action. OC treatment in transgenic 5xFAD mice reduced cerebral Aβ levels and inhibited neuroinflammation via dual inhibition of the NF-κB pathway and NLRP3 inflammasome. It is worth noting that OC displayed a greater anti-inflammatory profile than the phenolics of EVOO due to its inhibition of the RAGE/HMGB1 signaling pathway (77). This activity serves as an anti-inflammatory as it prevents downregulation of SNAP-25 and PSD-95. These are vital synaptic proteins in neurons that respond to the AβO continual presence (79). Furthermore, it prevents downregulation of astrocytic glutamate (GLT1) as well as glucose (GLUT1) transporters. Oleanolic acid protects brain synaptic function and provides metabolic support by counteracting neuroinflammatory signaling involving IL-6 and GFAP elevation. Another important mechanism is that it enhances amyloid-β clearance. OC enhances the efflux of Aβ across the blood-brain barrier (BBB) by regulating the levels of P-glycoprotein (P-gp) and LRP1. In cellular models using mouse brain endothelial cells as well as *in vivo* studies, it was shown that OC administration increased the brain efflux index of I-Aβ40 in C57BL/6 wild-type mice from 62.0 ± 3.0% to 79.9 ± 1.6% (80, 81). Further mechanistic studies in TgSwDI mice indicate that OC also activates the ApoE-dependent amyloid clearance pathway and increases Aβ-degrading enzymes, allowing a multifaceted reduction in cerebral amyloid burden (80). The efficacy of OC against tau pathology might also show its effective involvement against tau pathology seen in AD. Research in biochemistry indicates that OC or omega-3 fatty acid prevents the cross-linking of tau protein, which prevents the clotting of this protein. It is better to take the help of supplements for it (82).

A specific formulation may optimize the therapeutic potential of OC. Diets that contain olive oil enriched with oleanolic acid have been shown to not only inhibit the NLRP3 inflammasome, but they also activate the AMPK/ULK1 pathway and induce autophagic flux that restores the BBB effectively and also attenuates pathology linked with AD (83). Moreover, it has been found that EVOO shows a possible synergistic effect when used together with the standard AD drug donepezil. This combination therapy reduced Aβ burden, increased the expression of synaptic proteins, strengthened BBB integrity and reduced neuroinflammation. EVOO adjuncts may enhance conventional treatment via complementary non-cholinergic mechanisms (84).

### 6.2 Emerging evidence for Ligustroside

While OC is studied sufficiently, there are still many unknowns about ligustroside, which is a leading EVOO polyphenol. Nevertheless, there is ample proof that it is bioactive. The antioxidant and anti-inflammatory effects of ligustroside suggest it is effective on the immune-inflammatory factors of neurodegenerative diseases.

The study of the aging mouse model represents the most extensive assessment of the neuroprotective effect of ligustroside. Aged female NMRI mice (12-month-old) that received a diet with the ligustroside (50 mg/kg, 6.25 mg/kg body weight) for a period of 6 months showed an improvement in spatial working memory as compared to aged-control untreated mice. Furthermore, treating ligustroside resulted in restoration of ATP levels in the brain and a marked increase in lifespan (80). The ligustroside is thought to ameliorate age-related decline in energy metabolism, likely through modulation of mitochondrial bioenergetics rather than Aβ production pathways. This unique mechanism enhances the impact of OC, suggesting that the multi-component properties of EVOO phenolics could be optimally harnessed.

As per the recent evidence, it can be stated that OC can provide protection to the neurons through its actions relating to neuroinflammation, Aβ clearance, and tau pathology. Ligustroside, which has not been studied extensively, is a compound that appears to have unique impacts on mitochondrial performance and cognitive aging. In unison, these phenolics mark the medicinal worth of EVOO-derived compounds as well as warrant further studies to find out their worth in preventing or delaying AD.

## 7 Challenges and clinical translation prospects

In this review, we have summarized the efficacious roles of various EVOO polyphenols against the pathological processes of AD. These compounds target multiple fronts: hydroxytyrosol and oleuropein inhibit the aggregation of Aβ and tau; oleocanthal promotes Aβ clearance across the BBB; verbascoside and oleocanthal suppress neuroinflammation via NF-κB and NLRP3; hydroxytyrosol and others restore mitochondrial homeostasis and reduce oxidative stress; and several compounds promote neuroplasticity and synaptic function.

Despite compelling preclinical evidence, the translation of EVOO polyphenols into validated combinatorial therapies or preventive strategies for AD faces significant challenges. A major hurdle is the gap between animal models and human AD, particularly regarding disease complexity, progression, and species differences in metabolism and pharmacology. While epidemiological studies, such as those on the Mediterranean diet, suggest cognitive benefits, well-designed, large-scale, long-term human intervention trials specifically targeting EVOO polyphenols in AD prevention and management are still limited. Future clinical research must address critical questions regarding optimal dosing, formulation for enhanced bioavailability, treatment duration, and the identification of specific patient populations most likely to benefit. Furthermore, the synergistic effects of the EVOO polyphenol complex, as opposed to isolated compounds, need rigorous evaluation in humans. The safety profile of EVOO is excellent, which facilitates its consideration for long-term use; however, standardized extracts with defined polyphenol content are necessary for reproducible clinical outcomes.

In recent years, the impact of diet on human health has become a major research focus. Individuals are increasingly seeking simple, dietary means to prevent disease or support health. EVOO, a staple of the Mediterranean diet rich in natural polyphenols, is well-positioned to play an important role in this context. Future research should prioritize human studies that integrate biomarkers of AD pathology, neuroimaging, and cognitive assessments to firmly establish the role of EVOO and its bioactive polyphenols in combating Alzheimer’s disease.

## 8 Conclusion

The multifaceted pathology of Alzheimer’s disease necessitates a shift from single-target to multi-target therapeutic strategies. As detailed in this review, extra virgin olive oil polyphenols—including hydroxytyrosol, oleuropein, tyrosol, verbascoside, oleocanthal, and ligustroside—exert synergistic neuroprotection by concurrently modulating a network of key AD-related pathways. Their ability to inhibit Aβ and tau aggregation, enhance Aβ clearance, suppress chronic neuroinflammation, restore mitochondrial and metabolic function, reduce oxidative stress, and promote neurogenesis and synaptic plasticity underscores their significant potential. While challenges in clinical translation remain, the compelling preclinical evidence, coupled with the safety and dietary relevance of EVOO, positions these natural compounds as promising candidates for integrative approaches to delay the onset and slow the progression of AD. Future research should focus on validating these benefits in human trials and developing optimized formulations for clinical application.

## References

[1] Soria Lopez JA González HM Léger GC. Alzheimer’s disease. Handb Clin Neurol. (2019) 167:231–55. doi: 10.1016/b978-0-12-804766-8.00013-3 31753135

[2] Trejo-Lopez JA Yachnis AT Prokop S. Neuropathology of Alzheimer’s disease. Neurotherapeutics. (2022) 19:173–85. doi: 10.1007/s13311-021-01146-y, PMID: 34729690 PMC9130398

[3] Jarrott B. Tacrine: *in vivo* veritas. Pharmacol Res. (2017) 116:29–31. doi: 10.1016/j.phrs.2016.12.033, PMID: 28040533

[4] Marucci G Buccioni M Ben DD Lambertucci C Volpini R Amenta F. Efficacy of acetylcholinesterase inhibitors in Alzheimer’s disease. Neuropharmacology. (2021) 190:108352. doi: 10.1016/j.neuropharm.2020.108352, PMID: 33035532

[5] Chen YH Wang C Kurth T. Acetylcholinesterase inhibitors, Amd, and Alzheimer disease. JAMA Ophthalmol. (2024) 142:683–4. doi: 10.1001/jamaophthalmol.2024.1201, PMID: 38722653

[6] Noetzli M Eap CB. Pharmacodynamic, pharmacokinetic and pharmacogenetic aspects of drugs used in the treatment of Alzheimer’s disease. Clin Pharmacokinet. (2013) 52:225–41. doi: 10.1007/s40262-013-0038-9, PMID: 23408070

[7] McShane R Westby MJ Roberts E Minakaran N Schneider L Farrimond LE . Memantine for dementia. Cochrane Database Syst Rev. (2019) 3:Cd003154. doi: 10.1002/14651858.CD003154.pub6, PMID: 30891742 PMC6425228

[8] Buccellato FR D’Anca M Tartaglia GM Del Fabbro M Scarpini E Galimberti D. Treatment of Alzheimer’s disease: beyond symptomatic therapies. Int J Mol Sci. (2023) 24:13900. doi: 10.3390/ijms241813900 37762203 PMC10531090

[9] Kim AY Al Jerdi S MacDonald R Triggle CR. Alzheimer’s disease and its treatment-yesterday, today, and tomorrow. Front Pharmacol. (2024) 15:1399121. doi: 10.3389/fphar.2024.1399121, PMID: 38868666 PMC11167451

[10] Farihi A Bouhrim M Chigr F Elbouzidi A Bencheikh N Zrouri H . Exploring medicinal herbs’ therapeutic potential and molecular docking analysis for compounds as potential inhibitors of human acetylcholinesterase in Alzheimer’s disease treatment. Medicina (Kaunas). (2023) 59:1812. doi: 10.3390/medicina59101812, PMID: 37893530 PMC10608285

[11] Baranowska-Wójcik E Gajowniczek-Ałasa D Pawlikowska-Pawlęga B Szwajgier D. The potential role of phytochemicals in Alzheimer’s disease. Nutrients. (2025) 17:653. doi: 10.3390/nu17040653, PMID: 40004981 PMC11858096

[12] Gąsiorowski K Brokos JB Sochocka M Ochnik M Chojdak-Łukasiewicz J Zajączkowska K . Current and near-future treatment of Alzheimer’s disease. Curr Neuropharmacol. (2022) 20:1144–57. doi: 10.2174/1570159x19666211202124239, PMID: 34856906 PMC9886829

[13] Li Z Zhao T Shi M Wei Y Huang X Shen J . Polyphenols: natural food grade biomolecules for treating neurodegenerative diseases from a multi-target perspective. Front Nutr. (2023) 10:1139558. doi: 10.3389/fnut.2023.1139558 36925964 PMC10011110

[14] Vicente-Zurdo D Gómez-Mejía E Rosales-Conrado N León-González ME. A comprehensive analytical review of polyphenols: evaluating neuroprotection in Alzheimer’s disease. Int J Mol Sci. (2024) 25:5906. doi: 10.3390/ijms25115906, PMID: 38892094 PMC11173253

[15] Thawabteh AM Ghanem AW AbuMadi S Thaher D Jaghama W Karaman D . Promising natural remedies for Alzheimer’s disease therapy. Molecules. (2025) 30:922. doi: 10.3390/molecules30040922, PMID: 40005231 PMC11858286

[16] Aktary N Jeong Y Oh S Shin Y Sung Y Rahman M . Unveiling the therapeutic potential of natural products in Alzheimer’s disease: insights from in vitro, in vivo, and clinical studies. Front Pharmacol. (2025) 16:1601712. doi: 10.3389/fphar.2025.1601712, PMID: 40626308 PMC12230064

[17] Zivari-Ghader T Valioglu F Eftekhari A Aliyeva I Beylerli O Davran S . Recent progresses in natural based therapeutic materials for Alzheimer’s disease. Heliyon. (2024) 10:e26351. doi: 10.1016/j.heliyon.2024.e26351, PMID: 38434059 PMC10906329

[18] Bravo L. Polyphenols: chemistry, dietary sources, metabolism, and nutritional significance. Nutr Rev. (1998) 56:317–33. doi: 10.1111/j.1753-4887.1998.tb01670.x, PMID: 9838798

[19] Kalogeropoulos N Tsimidou MZ. Antioxidants in Greek virgin olive oils. Antioxidants. (2014) 3:387–413. doi: 10.3390/antiox3020387, PMID: 26784878 PMC4665486

[20] Gorzynik-Debicka M Przychodzen P Cappello F Kuban-Jankowska A Marino Gammazza A Knap N . Potential health benefits of olive oil and plant polyphenols. Int J Mol Sci. (2018) 19:686. doi: 10.3390/ijms19030686, PMID: 29495598 PMC5877547

[21] Leri M Bertolini A Stefani M Bucciantini M. Evoo polyphenols relieve synergistically autophagy dysregulation in a cellular model of Alzheimer’s disease. Int J Mol Sci. (2021) 22:7225. doi: 10.3390/ijms22137225, PMID: 34281279 PMC8267626

[22] Bucciantini M Leri M Nardiello P Casamenti F Stefani M. Olive polyphenols: antioxidant and anti-inflammatory properties. Antioxidants. (2021) 10:1044. doi: 10.3390/antiox10071044, PMID: 34209636 PMC8300823

[23] Fabiani R. Anti-cancer properties of olive oil secoiridoid phenols: a systematic review of *in vivo* studies. Food Funct. (2016) 7:4145–59. doi: 10.1039/c6fo00958a, PMID: 27713961

[24] Manna C D'Angelo S Migliardi V Loffredi E Mazzoni O Morrica P . Protective effect of the phenolic fraction from virgin olive oils against oxidative stress in human cells. J Agric Food Chem. (2002) 50:6521–6. doi: 10.1021/jf020565+, PMID: 12381144

[25] Visioli F Bellosta S Galli C. Oleuropein, the bitter principle of olives, enhances nitric oxide production by mouse macrophages. Life Sci. (1998) 62:541–6. doi: 10.1016/s0024-3205(97)01150-8, PMID: 9464466

[26] Wiseman SA Mathot JN de Fouw NJ Tijburg LB. Dietary non-tocopherol antioxidants present in extra virgin olive oil increase the resistance of low density lipoproteins to oxidation in rabbits. Atherosclerosis. (1996) 120:15–23. doi: 10.1016/0021-9150(95)05656-4, PMID: 8645356

[27] Owen RW Giacosa A Hull WE Haubner R Spiegelhalder B Bartsch H. The antioxidant/anticancer potential of phenolic compounds isolated from olive oil. Eur J Cancer. (2000) 36:1235–47. doi: 10.1016/s0959-8049(00)00103-9, PMID: 10882862

[28] Tripoli E Giammanco M Tabacchi G Di Majo D Giammanco S La Guardia M. The phenolic compounds of olive oil: structure, biological activity and beneficial effects on human health. Nutr Res Rev. (2005) 18:98–112. doi: 10.1079/nrr200495, PMID: 19079898

[29] Fleming HP Walter WM Jr Etchells JL. Antimicrobial properties of oleuropein and products of its hydrolysis from green olives. Appl Microbiol. (1973) 26:777–82. doi: 10.1128/am.26.5.777-782.1973, PMID: 4762397 PMC379901

[30] Federici F Bongi G. Improved method for isolation of bacterial inhibitors from oleuropein hydrolysis. Appl Environ Microbiol. (1983) 46:509–10. doi: 10.1128/aem.46.2.509-510.1983, PMID: 16346374 PMC239433

[31] Carluccio MA Siculella L Ancora MA Massaro M Scoditti E Storelli C . Olive oil and red wine antioxidant polyphenols inhibit endothelial activation: antiatherogenic properties of mediterranean diet phytochemicals. Arterioscler Thromb Vasc Biol. (2003) 23:622–9. doi: 10.1161/01.Atv.0000062884.69432.A0, PMID: 12615669

[32] Edgecombe SC Stretch GL Hayball PJ. Oleuropein, an antioxidant polyphenol from olive oil, is poorly absorbed from isolated perfused rat intestine. J Nutr. (2000) 130:2996–3002. doi: 10.1093/jn/130.12.2996, PMID: 11110859

[33] Robles-Almazan M Pulido-Moran M Moreno-Fernandez J Ramirez-Tortosa C Rodriguez-Garcia C Quiles JL . Hydroxytyrosol: bioavailability, toxicity, and clinical applications. Food Res Int. (2018) 105:654–67. doi: 10.1016/j.foodres.2017.11.053, PMID: 29433260

[34] Laghezza Masci V Bernini R Villanova N Clemente M Cicaloni V Tinti L . *In vitro* anti-proliferative and apoptotic effects of hydroxytyrosyl oleate on Sh-Sy5y human neuroblastoma cells. Int J Mol Sci. (2022) 23:12348. doi: 10.3390/ijms232012348, PMID: 36293207 PMC9604296

[35] Velotti F Bernini R. Hydroxytyrosol interference with inflammaging via modulation of inflammation and autophagy. Nutrients. (2023) 15:1774. doi: 10.3390/nu15071774, PMID: 37049611 PMC10096543

[36] Varghese N Werner S Grimm A Eckert A. Dietary mitophagy enhancer: a strategy for healthy brain aging? Antioxidants. (2020) 9:932. doi: 10.3390/antiox9100932, PMID: 33003315 PMC7600282

[37] Reutzel M Grewal R Silaidos C Zotzel J Marx S Tretzel J . Effects of Long-term treatment with a blend of highly purified olive secoiridoids on cognition and brain ATP levels in aged NMRI mice. Oxid Med Cell Longev. (2018) 2018:4070935. doi: 10.1155/2018/4070935, PMID: 30510619 PMC6232801

[38] D’Andrea G Ceccarelli M Bernini R Clemente M Santi L Caruso C . Hydroxytyrosol stimulates neurogenesis in aged dentate gyrus by enhancing stem and progenitor cell proliferation and neuron survival. FASEB J. (2020) 34:4512–26. doi: 10.1096/fj.201902643R 32027412

[39] Daccache A Lion C Sibille N Gerard M Slomianny C Lippens G . Oleuropein and derivatives from olives as tau aggregation inhibitors. Neurochem Int. (2011) 58:700–7. doi: 10.1016/j.neuint.2011.02.010, PMID: 21333710

[40] Wu L Velander P Liu D Xu B. Olive component oleuropein promotes Β-cell insulin secretion and protects Β-cells from amylin amyloid-induced cytotoxicity. Biochemistry. (2017) 56:5035–9. doi: 10.1021/acs.biochem.7b00199, PMID: 28829122

[41] Gallardo-Fernández M Hornedo-Ortega R Cerezo AB Troncoso AM García-Parrilla MC. Melatonin, protocatechuic acid and hydroxytyrosol effects on vitagenes system against alpha-synuclein toxicity. Food Chem Toxicol. (2019) 134:110817. doi: 10.1016/j.fct.2019.110817, PMID: 31521636

[42] Arunsundar M Shanmugarajan TS Ravichandran V. 3,4-Dihydroxyphenylethanol attenuates Spatio-cognitive deficits in an Alzheimer’s disease mouse model: modulation of the molecular signals in neuronal survival-apoptotic programs. Neurotox Res. (2015) 27:143–55. doi: 10.1007/s12640-014-9492-x, PMID: 25274193

[43] Ahamad J Toufeeq I Khan MA Ameen MSM Anwer ET Uthirapathy S . Oleuropein: a natural antioxidant molecule in the treatment of metabolic syndrome. Phytother Res. (2019) 33:3112–28. doi: 10.1002/ptr.6511, PMID: 31746508

[44] Micheli L Bertini L Bonato A Villanova N Caruso C Caruso M . Role of hydroxytyrosol and oleuropein in the prevention of aging and related disorders: focus on neurodegeneration, skeletal muscle dysfunction and gut microbiota. Nutrients. (2023) 15:1767. doi: 10.3390/nu15071767, PMID: 37049607 PMC10096778

[45] Marianetti M Pinna S Venuti A Liguri G. Olive polyphenols and bioavailable glutathione: promising results in patients diagnosed with mild Alzheimer’s disease. Alzheimers Dement. (2022) 8:e12278. doi: 10.1002/trc2.12278, PMID: 35310529 PMC8918095

[46] Leri M Chaudhary H Iashchishyn IA Pansieri J Svedružić ŽM Gómez Alcalde S . Natural compound from olive oil inhibits S100a9 amyloid formation and cytotoxicity: implications for preventing Alzheimer’s disease. ACS Chem Neurosci. (2021) 12:1905–18. doi: 10.1021/acschemneuro.0c00828, PMID: 33979140 PMC8291483

[47] Butt MS Tariq U Iahtisham Ul H Naz A Rizwan M. Neuroprotective effects of oleuropein: recent developments and contemporary research. J Food Biochem. (2021) 45:e13967. doi: 10.1111/jfbc.13967 34716610

[48] Abdallah IM Al-Shami KM Yang E Wang J Guillaume C Kaddoumi A. Oleuropein-rich olive leaf extract attenuates neuroinflammation in the Alzheimer’s disease mouse model. ACS Chem Neurosci. (2022) 13:1002–13. doi: 10.1021/acschemneuro.2c00005, PMID: 35263086

[49] Pantano D Luccarini I Nardiello P Servili M Stefani M Casamenti F. Oleuropein aglycone and polyphenols from olive mill waste water ameliorate cognitive deficits and neuropathology. Br J Clin Pharmacol. (2017) 83:54–62. doi: 10.1111/bcp.12993, PMID: 27131215 PMC5338135

[50] Martorell M Forman K Castro N Capó X Tejada S Sureda A. Potential therapeutic effects of oleuropein aglycone in Alzheimer’s disease. Curr Pharm Biotechnol. (2016) 17:994–1001. doi: 10.2174/1389201017666160725120656, PMID: 27455905

[51] Romero-Márquez JM Forbes-Hernández TY Navarro-Hortal MD Quirantes-Piné R Grosso G Giampieri F . Molecular mechanisms of the protective effects of olive leaf polyphenols against Alzheimer’s disease. Int J Mol Sci. (2023) 24:4353. doi: 10.3390/ijms24054353, PMID: 36901783 PMC10001635

[52] Reyes-Corral M Gil-González L González-Díaz Á Tovar-Luzón J Ayuso MI Lao-Pérez M . Pretreatment with oleuropein protects the neonatal brain from hypoxia-ischemia by inhibiting apoptosis and neuroinflammation. J Cereb Blood Flow Metab. (2025) 45:717–34. doi: 10.1177/0271678x241270237, PMID: 39157939 PMC12314356

[53] Gonçalves M Costa M Paiva-Martins F Silva P. Olive oil industry by-products as a novel source of biophenols with a promising role in Alzheimer disease prevention. Molecules. (2024) 29:4841. doi: 10.3390/molecules29204841, PMID: 39459209 PMC11510978

[54] Luccarini I Ed Dami T Grossi C Rigacci S Stefani M Casamenti F. Oleuropein aglycone counteracts Aβ42 toxicity in the rat brain. Neurosci Lett. (2014) 558:67–72. doi: 10.1016/j.neulet.2013.10.062, PMID: 24211687

[55] Rigacci S Guidotti V Bucciantini M Parri M Nediani C Cerbai E . Oleuropein aglycon prevents cytotoxic amyloid aggregation of human amylin. J Nutr Biochem. (2010) 21:726–35. doi: 10.1016/j.jnutbio.2009.04.010, PMID: 19616928

[56] Diomede L Rigacci S Romeo M Stefani M Salmona M. Oleuropein aglycone protects transgenic *C. elegans* strains expressing Aβ42 by reducing plaque load and motor deficit. PLoS One. (2013) 8:e58893. doi: 10.1371/journal.pone.0058893, PMID: 23520540 PMC3592812

[57] Palazzi L Bruzzone E Bisello G Leri M Stefani M Bucciantini M . Oleuropein aglycone stabilizes the monomeric α-synuclein and favours the growth of non-toxic aggregates. Sci Rep. (2018) 8:8337. doi: 10.1038/s41598-018-26645-5, PMID: 29844450 PMC5974307

[58] Luccarini I Grossi C Rigacci S Coppi E Pugliese AM Pantano D . Oleuropein aglycone protects against pyroglutamylated-3 amyloid-ß toxicity: biochemical, epigenetic and functional correlates. Neurobiol Aging. (2015) 36:648–63. doi: 10.1016/j.neurobiolaging.2014.08.029, PMID: 25293421

[59] Shibani F Sahamsizadeh A Fatemi I Allahtavakoli M Hasanshahi J Rahmani M . Effect of oleuropein on morphine-induced hippocampus neurotoxicity and memory impairments in rats. Naunyn Schmiedebergs Arch Pharmacol. (2019) 392:1383–91. doi: 10.1007/s00210-019-01678-3, PMID: 31236657

[60] Plotnikov MB Plotnikova TM. Tyrosol as a neuroprotector: strong effects of a “weak” antioxidant. Curr Neuropharmacol. (2021) 19:434–48. doi: 10.2174/1570159x18666200507082311, PMID: 32379590 PMC8206466

[61] Wang W Du L Wei Q Lu M Xu D Li Y. Synthesis and health effects of phenolic compounds: a focus on tyrosol, hydroxytyrosol, and 3,4-dihydroxyacetophenone. Antioxidants. (2025) 14:476. doi: 10.3390/antiox14040476, PMID: 40298838 PMC12024331

[62] Rodríguez-Morató J Boronat A Serreli G Enríquez L Gomez-Gomez A Pozo OJ . Effects of wine and tyrosol on the lipid metabolic profile of subjects at risk of cardiovascular disease: potential cardioprotective role of ceramides. Antioxidants. (2021) 10:1679. doi: 10.3390/antiox10111679, PMID: 34829550 PMC8614856

[63] Sun X Zhao M Wang X Sun Y Li J Zhang Y . Tyrosol ameliorates depressive-like behavior and hippocampal damage in perimenopausal depression rats by inhibiting oxidative stress and thyroid dysfunction. Neurosci Lett. (2025) 859-861:138266. doi: 10.1016/j.neulet.2025.138266, PMID: 40383458

[64] Taniguchi K Yamamoto F Arai T Yang J Sakai Y Itoh M . Tyrosol reduces amyloid-β oligomer neurotoxicity and alleviates synaptic, oxidative, and cognitive disturbances in Alzheimer’s disease model mice. J Alzheimers Dis. (2019) 70:937–52. doi: 10.3233/jad-190098, PMID: 31227651

[65] St-Laurent-Thibault C Arseneault M Longpré F Ramassamy C. Tyrosol and hydroxytyrosol, two main components of olive oil, protect N2a cells against amyloid-Β-induced toxicity. Involvement of the Nf-κB signaling. Curr Alzheimer Res. (2011) 8:543–51. doi: 10.2174/156720511796391845, PMID: 21605049

[66] Kwon MJ Lee JW Kim KS Chen H Cui CB Lee GW . The influence of tyrosol-enriched *Rhodiola Sachalinensis* extracts bioconverted by the mycelium of Bovista Plumbe on scopolamine-induced cognitive, behavioral, and physiological responses in mice. Molecules. (2022) 27:4455. doi: 10.3390/molecules27144455, PMID: 35889329 PMC9324053

[67] Chen S Liu H Wang S Jiang H Gao L Wang L . The neuroprotection of verbascoside in Alzheimer’s disease mediated through mitigation of neuroinflammation via blocking NF-κB-P65 signaling. Nutrients. (2022) 14:1417. doi: 10.3390/nu14071417, PMID: 35406030 PMC9003273

[68] Su Y Liu N Sun R Ma J Li Z Wang P . Radix Rehmanniae Praeparata (Shu Dihuang) exerts neuroprotective effects on ICV-STZ-induced Alzheimer’s disease mice through modulation of INSR/IRS-1/AKT/GSK-3β signaling pathway and intestinal microbiota. Front Pharmacol. (2023) 14:1115387. doi: 10.3389/fphar.2023.1115387, PMID: 36843923 PMC9945319

[69] Wang C Ye H Zheng Y Qi Y Zhang M Long Y . Phenylethanoid glycosides of cistanche improve learning and memory disorders in app/Ps1 mice by regulating glial cell activation and inhibiting TLR4/NF-κB signaling pathway. NeuroMolecular Med. (2023) 25:75–93. doi: 10.1007/s12017-022-08717-y, PMID: 35781783

[70] Peng XM Gao L Huo SX Liu XM Yan M. The mechanism of memory enhancement of acteoside (verbascoside) in the senescent mouse model induced by a combination of D-gal and Alcl3. Phytother Res. (2015) 29:1137–44. doi: 10.1002/ptr.5358, PMID: 25900087

[71] Gao L Peng XM Huo SX Liu XM Yan M. Memory enhancement of acteoside (verbascoside) in a senescent mice model induced by a combination of D-gal and AlCl3. Phytother Res. (2015) 29:1131–6. doi: 10.1002/ptr.5357, PMID: 25900014

[72] Wang C Cai X Wang R Zhai S Zhang Y Hu W . Neuroprotective effects of verbascoside against Alzheimer’s disease via the relief of endoplasmic reticulum stress in Aβ-exposed U251 cells and APP/PS1 mice. J Neuroinflammation. (2020) 17:309. doi: 10.1186/s12974-020-01976-1, PMID: 33070776 PMC7570123

[73] Zhou ZH Xing HY Liang Y Gao J Liu Y Zhang T . Molecular mechanism of verbascoside in promoting acetylcholine release of neurotransmitter. Zhongguo Zhong Yao Za Zhi. (2025) 50:335–48. doi: 10.19540/j.cnki.cjcmm.20240802.702, PMID: 39929615

[74] Korshavn KJ Jang M Kwak YJ Kochi A Vertuani S Bhunia A . Reactivity of metal-free and metal-associated amyloid-β with glycosylated polyphenols and their esterified derivatives. Sci Rep. (2015) 5:17842. doi: 10.1038/srep17842, PMID: 26657338 PMC4674742

[75] Tajmim A Cuevas-Ocampo AK Siddique AB Qusa MH King JA Abdelwahed KS . (−)-Oleocanthal nutraceuticals for Alzheimer’s disease amyloid pathology: novel oral formulations, therapeutic, and molecular insights in 5xfad transgenic mice model. Nutrients. (2021) 13:1702. doi: 10.3390/nu13051702, PMID: 34069842 PMC8157389

[76] Yang E Wang J Woodie LN Greene MW Kaddoumi A. Oleocanthal ameliorates metabolic and behavioral phenotypes in a mouse model of Alzheimer’s disease. Molecules. (2023) 28:5592. doi: 10.3390/molecules28145592, PMID: 37513464 PMC10385639

[77] Abdallah IM Al-Shami KM Alkhalifa AE Al-Ghraiybah NF Guillaume C Kaddoumi A. Comparison of oleocanthal-low Evoo and oleocanthal against amyloid-β and related pathology in a mouse model of Alzheimer’s disease. Molecules. (2023) 28:1249. doi: 10.3390/molecules28031249, PMID: 36770920 PMC9921117

[78] Zupo R Castellana F Panza F Solfrizzi V Lozupone M Tardugno R . Alzheimer’s disease may benefit from olive oil polyphenols: a systematic review on preclinical evidence supporting the effect of oleocanthal on amyloid-β load. Curr Neuropharmacol. (2025) 23:1249–59. doi: 10.2174/011570159x327650241021115228, PMID: 39482909 PMC12307993

[79] Batarseh YS Mohamed LA Al Rihani SB Mousa YM Siddique AB El Sayed KA . Oleocanthal ameliorates amyloid-β oligomers’ toxicity on astrocytes and neuronal cells: in vitro studies. Neuroscience. (2017) 352:204–15. doi: 10.1016/j.neuroscience.2017.03.059, PMID: 28392295 PMC5504696

[80] Qosa H Batarseh YS Mohyeldin MM El Sayed KA Keller JN Kaddoumi A. Oleocanthal enhances amyloid-β clearance from the brains of TgSwDI mice and *in vitro* across a human blood-brain barrier model. ACS Chem Neurosci. (2015) 6:1849–59. doi: 10.1021/acschemneuro.5b00190, PMID: 26348065 PMC4653763

[81] Abuznait AH Qosa H Busnena BA El Sayed KA Kaddoumi A. Olive-oil-derived oleocanthal enhances β-amyloid clearance as a potential neuroprotective mechanism against Alzheimer’s disease: *in vitro* and *in vivo* studies. ACS Chem Neurosci. (2013) 4:973–82. doi: 10.1021/cn400024q, PMID: 23414128 PMC3689195

[82] Monti MC Margarucci L Riccio R Casapullo A. Modulation of tau protein fibrillization by oleocanthal. J Nat Prod. (2012) 75:1584–8. doi: 10.1021/np300384h, PMID: 22988908

[83] Al Rihani SB Darakjian LI Kaddoumi A. Oleocanthal-rich extra-virgin olive oil restores the blood-brain barrier function through NLRP3 inflammasome inhibition simultaneously with autophagy induction in TgSwDI mice. ACS Chem Neurosci. (2019) 10:3543–54. doi: 10.1021/acschemneuro.9b00175, PMID: 31244050 PMC6703911

[84] Batarseh YS Kaddoumi A. Oleocanthal-rich extra-virgin olive oil enhances donepezil effect by reducing amyloid-β load and related toxicity in a mouse model of Alzheimer’s disease. J Nutr Biochem. (2018) 55:113–23. doi: 10.1016/j.jnutbio.2017.12.006, PMID: 29413486 PMC5936648

[85] Wang W Jing T Yang X He Y Wang B Xiao Y . Hydroxytyrosol regulates the autophagy of vascular adventitial fibroblasts through the SIRT1-mediated signaling pathway. Can J Physiol Pharmacol. (2018) 96:88–96. doi: 10.1139/cjpp-2016-0676, PMID: 28772080

[86] Qin C Hu S Zhang S Zhao D Wang Y Li H . Hydroxytyrosol acetate improves the cognitive function of APP/PS1 transgenic mice in ERβ-dependent manner. Mol Nutr Food Res. (2021) 65:e2000797. doi: 10.1002/mnfr.202000797, PMID: 33296142

